# Supplementary material for: Unique Phase Transition of Exogenous Fusion Elastin-like Polypeptides in the Solution Containing Polyethylene Glycol
Source: Int J Mol Sci. 2019 Jul 20;20(14):3560. doi: 10.3390/ijms20143560 (PMC6678693; doi:10.3390/ijms20143560)
Supplement: Supplementary file 1 [file ijms-20-03560-s001.pdf]

# Supplementary Materials

## Unique Phase Transition of Exogenous Fusion Elastin-like Polypeptides in the Solution Containing Polyethylene Glycol

**Zhongqi Ge, Ziyang Xiong, Dandan Zhang, Xialan Li * and Guangya Zhang ***

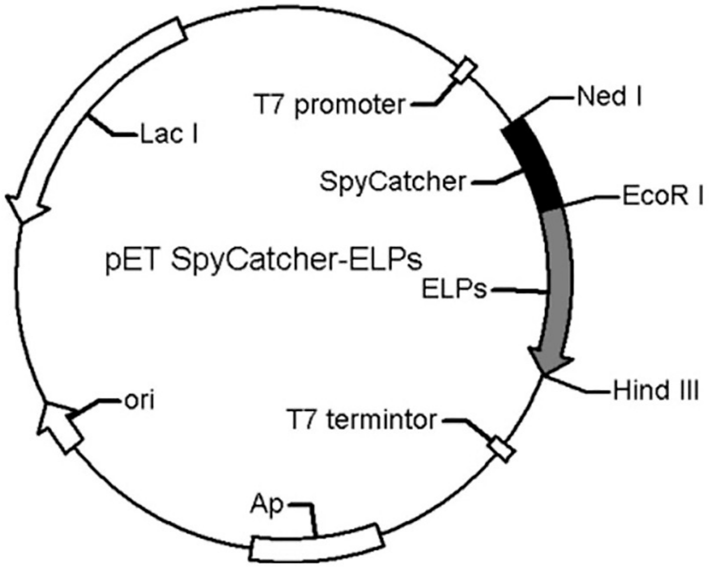


**Figure S1.** Plasmid mapping of expression vector of E-C (SpyCatcher-$ELPs_{40}$)

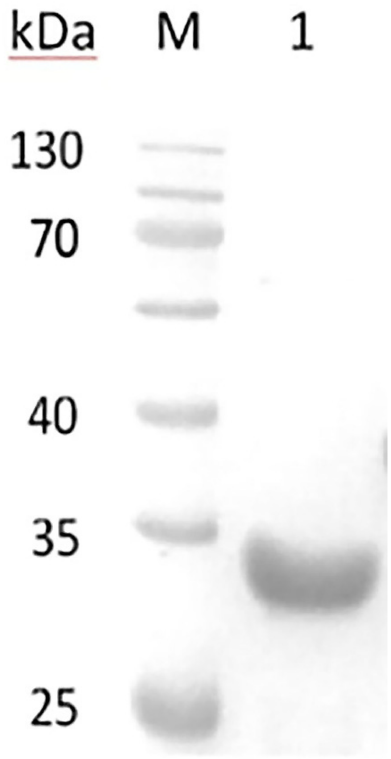


**Figure S2.** The SDS-PAGE of E-C. M: molecular mass marker; lane 1: E-C.

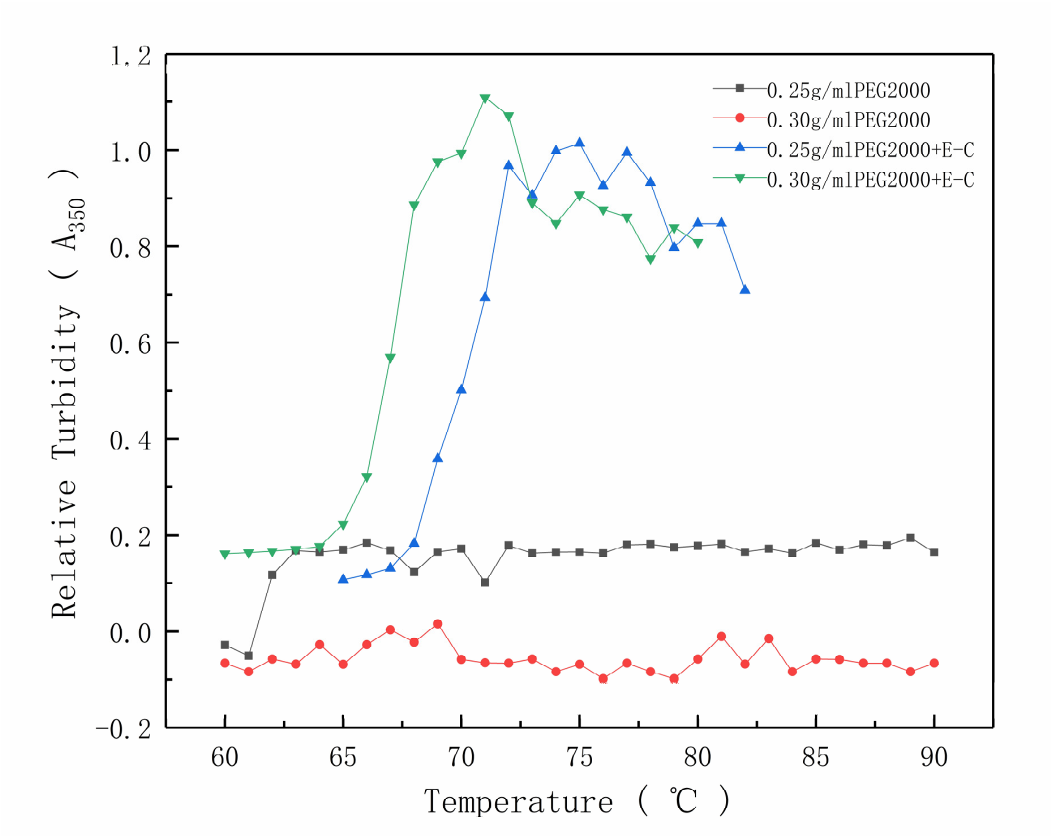


**Figure S3.** Effects of PEG2000 on the phase transitions of E-C in 0.3mol/L Na2CO3 solution.

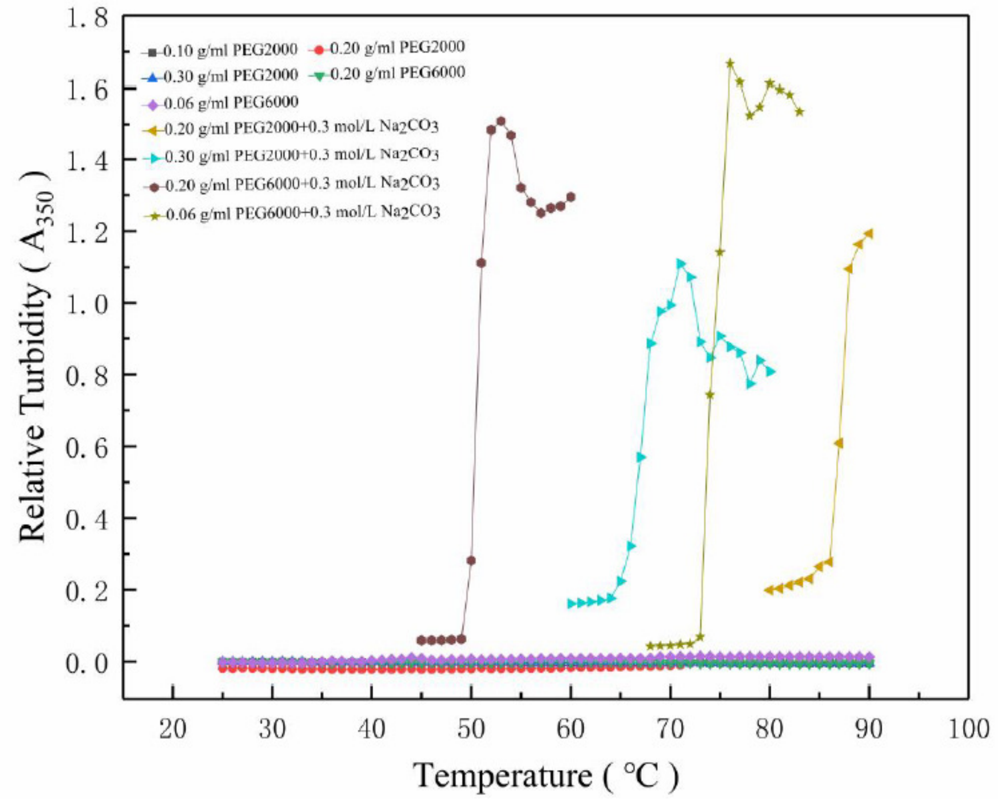


**Figure S4.** Effects of Na2CO3 on the phase transitions of E-C in PEG2000 solution.

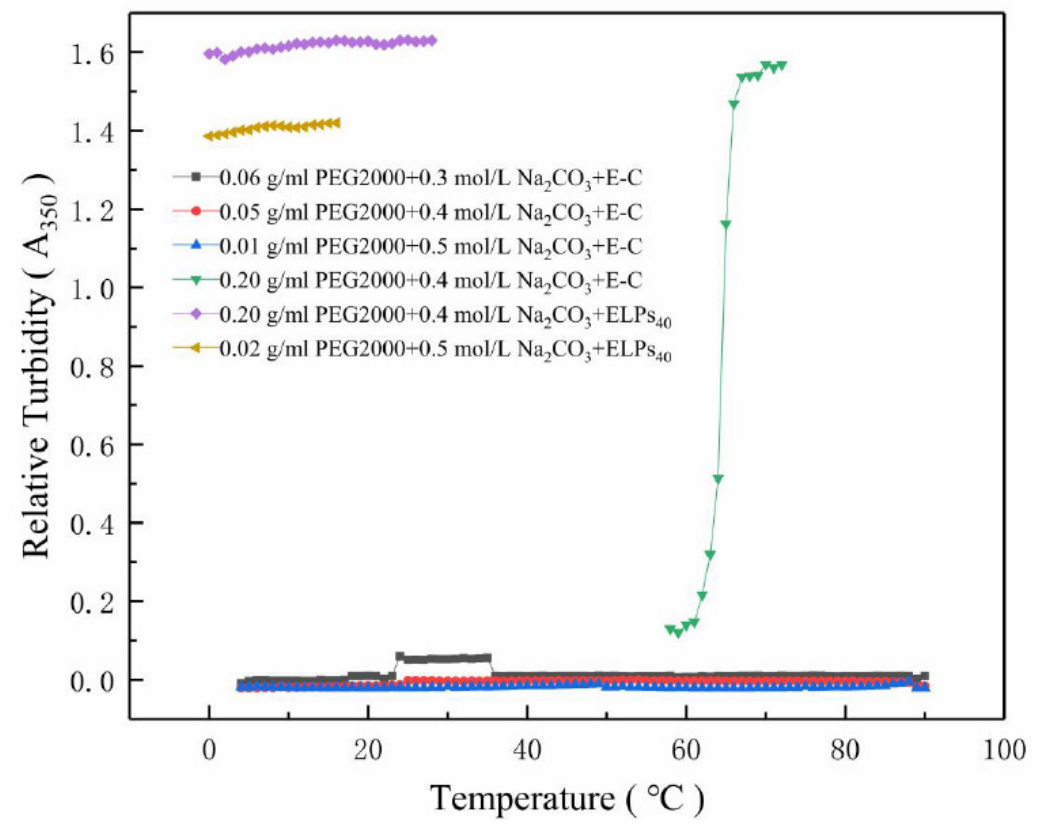


**Figure S5.** Effects of PEG/Na2CO3 concentration on phase transitions of ELPs40 and E-C.
